# Semi-Device-Independent Randomness Expansion Using *n*→1 Parity-Oblivious Quantum Random Access Codes

**DOI:** 10.3390/e27070696

**Published:** 2025-06-28

**Authors:** Xunan Wang, Xu Chen, Mengke Xu, Wanglei Mi, Xiao Chen

**Affiliations:** 1 College of Information Engineering, China Jiliang University, Hangzhou 310018, China; xunan@cjlu.edu.cn (X.W.); xmk22@cjlu.edu.cn (M.X.); 22h034160135@cjlu.edu.cn (W.M.); 2 College of Computer Science and Technology, Nanjing University of Aeronautics and Astronautics, Nanjing 211106, China; bx2416012@nuaa.edu.cn

**Keywords:** Bell inequality, randomness expansion, parity-obliviousness, min-entropy

## Abstract

Quantum mechanics enables the generation of genuine randomness through its intrinsic indeterminacy. In device-independent (DI) and semi-device-independent (SDI) frameworks, randomness generation protocols can further ensure that the output remains secure and unaffected by internal device imperfections, with certification grounded in violations of generalized Bell inequalities. In this work, we propose an SDI randomness expansion protocol using n→1 parity-oblivious quantum random access code (PO-QRAC), where the presence of true quantum randomness is certified through the violation of a two-dimensional quantum witness. For various values of *n*, we derive the corresponding maximal expected success probabilities. Notably, for n=4, the expected success probability obtained under our protocol exceeds the upper bound reported in prior work. Furthermore, we establish an analytic relationship between the certifiable min-entropy and the quantum witness value, and demonstrate that, for a fixed witness value, PO-QRAC–based protocols certify more randomness than those based on standard QRACs. Among all configurations satisfying the parity-obliviousness constraint, the protocol based on the 3→1 PO-QRAC achieves optimal randomness expansion performance.

## 1. Introduction

Random numbers serve as indispensable resources in various technological domains, including cryptography, secure computation, and quantum protocols [1]. In classical cryptographic frameworks, foundational protocols such as the Data Encryption Standard (DES) and Rivest–Shamir–Adleman (RSA) cryptosystems require random bit generation for cryptographic key establishment. In quantum cryptography, the BB84 quantum key distribution protocol relies critically on perfect random bits for secure basis selection and state encoding [2]. However, conventional random number generators (RNGs) produce only pseudorandom sequences whose security fundamentally relies on trust assumptions regarding device integrity [3]. Quantum mechanics provides a solution through its inherent non-determinism, enabling provably unpredictable randomness generation [4]. Consequently, device-independent (DI) and semi-device-independent (SDI) quantum RNGs have emerged as transformative paradigms in quantum information science [5,6,7,8].

The DI framework, formally established by Ac’in and Colbeck [9,10], guarantees security based solely on observed measurement statistics (e.g., Bell inequality violations) without device characterization. In contrast, SDI protocols relax these requirements by assuming prior knowledge of the system dimension while remaining agnostic to other device specifications [11]. Notably, the dimensionality of quantum systems can be experimentally determined through quantum dimension witnessing protocols [12,13]. This hierarchy of trust assumptions enables flexible implementations balancing security and practicality for next-generation randomness expansion protocols. Randomness expansion is a protocol that utilizes a small amount of initial randomness to generate a larger sequence of certified random numbers. In recent years, significant progress has been made in both DI and SDI frameworks. In the DI framework, pioneering work includes Colbeck’s genuine randomness expansion protocols based on GHZ tests for different randomness sources [14,15], and Pironio et al.’s protocol utilizing Bell inequality violations for randomness certification [16]. Coudron et al. presented a method achieving unbounded randomness expansion [17]. The development of SDI protocols has opened new research directions. Li et al. first proposed quantum random access code (QRAC)-based randomness expansion protocols in the SDI framework, rigorously proving that perfect random seeds can generate fresh randomness [18,19]. Zhou et al. subsequently extended this work by investigating protocols with partially free randomness sources under the SDI framework [20,21,22,23].

QRAC is a crucial tool in studying SDI quantum randomness expansion protocols. The study of QRACs in higher-dimensional Hilbert spaces and sequential QRACs have propelled the development of multi-party quantum randomness expansion protocols [24,25,26,27,28,29]. As a specialized variant of QRAC, parity-oblivious QRAC (PO-QRAC) introduces a critical constraint: the encoded quantum states must conceal parity information of the input classical bits, thereby addressing vulnerabilities in conventional QRACs that inadvertently leak global properties such as parity checks [30]. In a conventional n→1 RAC, Alice can effectively leak one of her input bits in clear form by embedding it directly into the transmitted state, allowing Bob to recover that bit with certainty. To eliminate this trivial information pathway, the parity-oblivious constraint demands that no parity of Alice’s input string may be learned from the communicated quantum states [31]. This requirement ensures that, regardless of Bob’s measurement strategy, the transmitted system carries no information about any parity bit—thereby precluding any classical “backdoor” and sharpening the focus on genuinely nonclassical, preparation-contextual advantages [32].

In quantum randomness expansion protocols, employing PO-QRACs prevents collusion among the parties and thereby enables the extraction of greater amounts of certifiable randomness. We introduce a randomness expansion based on the n→1 PO-QRAC and, for n=2,3,4, explicitly construct the corresponding two-dimensional quantum witnesses that certify quantum advantage. Finally, we derive an analytic relation between the quantum witness and the certifiable min-entropy, quantifying how the degree of witness violation translates into fresh randomness.

The remainder of this paper is organized as follows. In Section 2, we present the formal model of the proposed n→1 PO-QRAC protocol and define the parity-obliviousness constraint. Section 3 derives the optimal classical success probability under this model and identifies the best parity-oblivious classical codes. In Section 4, we compute the maximum expected success probability in the quantum scenario for each *n* and verify when a quantum witness violation occurs. Section 5 analyzes the relationship between quantum witness and the certifiable randomness in the resulting randomness-expansion protocol, comparing our PO-QRAC–based schemes against standard QRAC–based approaches. Finally, we present our conclusion in Section 6.

## 2. Model Description

**Figure 1:**
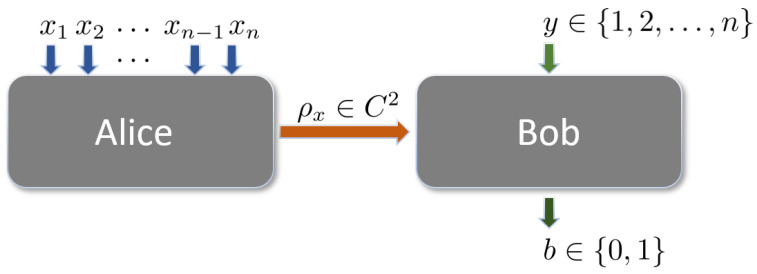
Semi-device-independent randomness expansion using n→1 QRAC involves two parties, Alice and Bob. Alice encodes a quantum state $\rho_{x}$ based on her input $x=\left\{x_{1},x_{2},\ldots ,x_{n}\right\}\in {\left\{0,1\right\}}^{n}$. Bob subsequently performs a quantum measurement on $\rho_{x}$ according to his input y∈{1,2,…,n}. The outcome of Bob’s measurement is a single bit b∈{0,1}, which serves as the model’s output. The implementation employs two protected black-box devices residing in the same secure space. The protocol operates under a semi-device-independent framework, where the internal workings of the devices are unknown, but the dimensions of the quantum systems are certified.

We begin by introducing the SDI randomness expansion model. The SDI framework requires that the quantum system be entanglement-free and imposes no assumptions on any parameters beyond the Hilbert space dimensionality. Our model is based on the n→1 QRAC in a two-dimensional Hilbert space $C^{2}$, which comprises two black-boxes: the preparation party (Alice) and the measurement party (Bob). As shown in Figure 1, Alice randomly selects a bit string $x=\left\{x_{1},x_{2},\ldots ,x_{n}\right\}\in {\left\{0,1\right\}}^{n}$, encodes it into a quantum state $\rho_{x}$, and transmits it to Bob. Upon receiving $\rho_{x}$, Bob performs a measurement defined by the POVM measurement ${\left\{{\hat{M}}_{y}^{b}\right\}}_{b=0}^{1}$ where y∈{1,…,n}, and subsequently outputs the measurement result b∈{0,1}.

Through multiple repetitions of the procedure, we evaluate the expected success probability

(1) $$E=\frac{1}{n\cdot 2^{n}}\sum_{x\in {\left\{0,1\right\}}^{n}}\sum_{y=1}^{n}p\left(b=x_{y}|x,y\right),$$

where success is defined as the event that Bob employs the y-th measurement ${\left\{{\hat{M}}_{y}^{b}\right\}}_{b=0}^{1}$ and obtains an outcome *b* matching the *y*-th bit of the input string *x*. We construct the two-dimensional quantum witness using the expected success probability.

The expected success probabilities, denoted as $E_{c}$ (classical) and $E_{q}$ (quantum), characterize the performance bounds in the respective scenarios. When the experimentally observed success probability $\hat{E}$ exceeds the classical upper bound $E_{c}^{\max}$, this provides a statistical proof of the system’s ability to generate certified quantum randomness. The output sequence {b} can then undergo quantum-proof entropy distillation to extract randomness that is information-theoretically secure. In this paper, we use the min-entropy function to quantify the randomness:

(2) $$H_{\infty }\left(B|X,Y\right)=-\log_{2}\left[\max_{x,y,b}p\left(b|x,y\right)\right].$$

We now introduce the concept of PO-QRAC in detail. The parity-obliviousness constraint enforces that Bob cannot obtain any parity information about the input bit string $x\in {\left\{0,1\right\}}^{n}$. Following reference [30], we define the parity-oblivious constraint set as

(3) $$S_{n}:=\left\{S|S=\left\{S_{1},S_{2},\ldots ,S_{n}\right\}\in {\left\{0,1\right\}}^{n},\sum_{i}^{n}S_{i}⩾2\right\}.$$

This leads to the quantum state constraint:

(4) $$\forall S\in S_{n}:\;\sum_{x\in {\left\{0,1\right\}}^{n}}{\left(-1\right)}^{{⨁}_{i}S_{i}x_{i}}\rho_{x}=0,$$

where ⨁ denotes modulo-2 summation.

## 3. The Maximum Expected Success Probabilities for the n→1 PO-RACs

In this section, we analyze the maximum expected success probability $E_{c}^{\max}$ for classical n→1 parity-oblivious random access codes (PO-RAC). For the classical standard n→1 RAC, the optimal encoding scheme $E:{\left\{0,1\right\}}^{n}\to \left\{0,1\right\}$ is given by the Hamming threshold function:

(5) $$E\left(x\right)=\left\{\begin{array}{cc} 0, & \mathrm{wt}(x)⩽\lfloor n/2\rfloor ,\\ 1, & \mathrm{wt}(x)>\lfloor n/2\rfloor , \end{array}\right.$$

where wt(x) denotes the Hamming weight of *x*. The corresponding optimal decoding scheme is characterized by the identity decoding strategy. According to [33], the maximum expected success probability for classical standard n→1 RAC is given by

(6) $$E_{c}^{\max}=\frac{1}{2}+\frac{1}{2^{n}}\left(\begin{array}{c} n-1\\ \left\lfloor (n-1)/2\right\rfloor \end{array}\right).$$

The incorporation of the parity-obliviousness constraint necessitates modifications to the optimal encoding scheme for standard n→1 RAC. For n=2, the parity-oblivious set is defined as $S_{2}=\left\{11\right\}$. This imposes the following linear constraint on the encoding function: E(00)+E(11)=E(01)+E(10). A valid resolution satisfying this constraint is E(00)=E(01)=0, E(10)=E(11)=1. Remarkably, this constrained encoding preserves the maximum expected success probability $E_{c}^{\max}=3/4$, identical to the standard 2→1 RAC.

For n>2, the parity-obliviousness constraint alters the maximum expected success probability of RACs compared to their standard counterparts.

In the case of n=3, the parity-oblivious set is defined as $S_{3}=\left\{011,101,110,111\right\}$, corresponding to all non-trivial parity functions over three-bit strings. To satisfy this constraint while maintaining near-optimal performance, we refine the encoding scheme in Equation (5) through strategic input reclassification, i.e., E(011)=0 and E(100)=1, while preserving the Hamming weight threshold rule for other inputs. The computational result demonstrates that the 3→1 PO-RAC achieves a maximum expected success probability of 2/3, which is less than the $E_{c}^{\max}$ of the standard 3→1 RAC.

In the case of n=4, the parity-oblivious set is defined as $S_{4}=\{0011,0101,0110,$0111,1001,1010,1011,1100,1101,1110,1111}. To reconcile this constraint with near-optimal performance, we encode the four-bit strings based on the value of the leading bit $x_{1}$. Then, the maximum expected success probability for the 4→1 PO-RAC is 5/8.

## 4. The Maximum Expected Success Probabilities for the n→1 PO-QRACs

This section establishes the theoretical maximum of the expected success probabilities for n→1 PO-QRACs. Within the quantum information framework, the encoding states employed in our protocol must rigorously adhere to the parity-oblivious constraint, ensuring that no measurable information about parity correlations can be extracted through quantum measurements.

For n=2, the parity-oblivious constraint manifests as $\rho_{00}+\rho_{11}=\rho_{01}+\rho_{10}$. Remarkably, the optimal standard 2→1 QRAC satisfies this condition through its symmetric encoding. We implement the following quantum scheme:

(7) $$\rho_{00}=|+\rangle \langle +|,\rho_{11}=|-\rangle \langle -|,\rho_{01}=|0\rangle \langle 0|,\rho_{10}=|1\rangle \langle 1|,$$

and

(8) $$\begin{array}{c} {\hat{M}}_{1}^{0}=\left(\begin{array}{cc} \frac{1}{2}+\frac{\sqrt{2}}{4} & \frac{\sqrt{2}}{4}\\ \frac{\sqrt{2}}{4} & \frac{1}{2}-\frac{\sqrt{2}}{4} \end{array}\right),\;{\hat{M}}_{1}^{1}=\left(\begin{array}{cc} \frac{1}{2}-\frac{\sqrt{2}}{4} & -\frac{\sqrt{2}}{4}\\ -\frac{\sqrt{2}}{4} & \frac{1}{2}+\frac{\sqrt{2}}{4} \end{array}\right), \end{array}$$

(9) $$\begin{array}{c} {\hat{M}}_{2}^{0}=\left(\begin{array}{cc} \frac{1}{2}-\frac{\sqrt{2}}{4} & \frac{\sqrt{2}}{4}\\ \frac{\sqrt{2}}{4} & \frac{1}{2}+\frac{\sqrt{2}}{4} \end{array}\right),\;{\hat{M}}_{2}^{1}=\left(\begin{array}{cc} \frac{1}{2}+\frac{\sqrt{2}}{4} & -\frac{\sqrt{2}}{4}\\ -\frac{\sqrt{2}}{4} & \frac{1}{2}-\frac{\sqrt{2}}{4} \end{array}\right). \end{array}$$

We obtain the expected success probability as follows:

(10) $$\begin{array}{cc} E_{q} & =\frac{1}{8}\sum_{x\in {\left\{0,1\right\}}^{2}}\sum_{y=1}^{2}\mathrm{tr}\left(\rho_{x}{\hat{M}}_{y}^{x_{y}}\right)\\ \; & =\frac{1}{2}+\frac{\sqrt{2}}{4}. \end{array}$$

This scheme is also optimal for 2→1 PO-QRAC, establishing the maximum expected success probability as $1/2+\sqrt{2}/4$.

For n=3, the parity-oblivious constraint corresponding to S=011 is satisfied if $\rho_{000}+\rho_{011}+\rho_{100}+\rho_{111}=\rho_{001}+\rho_{010}+\rho_{101}+\rho_{110}$. Similarly, three conditions can be obtained for S=101, S=110, and S=111. We represent the pure quantum state $\rho_{x}$ as a linear combination of Pauli matrices:

(11) $$\rho_{x}=\frac{1}{2}\left(I+r_{x}\cdot \sigma \right),$$

where $r_{x}$ is the Bloch vector for $\rho_{x}$, and $\sigma =\{\sigma_{x},\sigma_{y},\sigma z\}$. Each pure state corresponds to a Bloch vector on the unit sphere, enabling the success probability to be expressed in terms of vector inner products. To maximize the expected success probability, the Bloch vectors must satisfy the antipodal condition: $r_{x}$ = $-r_{\bar{x}}$, where $\bar{x}$ denotes the bitwise negation of *x*. The parity-oblivious constraint translates into solving the following system of equations:

(12) $$\begin{array}{c} r_{000}+r_{011}+r_{100}+r_{111}=r_{001}+r_{010}+r_{101}+r_{110}, \end{array}$$

(13) $$\begin{array}{c} r_{000}+r_{010}+r_{101}+r_{111}=r_{001}+r_{011}+r_{100}+r_{110}, \end{array}$$

(14) $$\begin{array}{c} r_{000}+r_{001}+r_{110}+r_{111}=r_{010}+r_{011}+r_{100}+r_{101}, \end{array}$$

(15) $$\begin{array}{c} r_{000}+r_{011}+r_{101}+r_{110}=r_{001}+r_{010}+r_{100}+r_{111}. \end{array}$$

Given the antipodal condition, we only need to consider Equation (15), reducing the constraint to $r_{000}+r_{011}=r_{001}+r_{010}$. The Bloch vector parameterization for quantum state preparation in the 3→1 PO-QRAC is expressed as

(16) $$\begin{array}{c} r_{000}=\left(\sin\theta_{1}\cos\varphi_{1},\sin\theta_{1}\sin\varphi_{1},\cos\theta_{1}\right),r_{001}=\left(\sin\theta_{2}\cos\varphi_{2},\sin\theta_{2}\sin\varphi_{2},\cos\theta_{2}\right), \end{array}$$

(17) $$\begin{array}{c} r_{010}=\left(\sin\theta_{3}\cos\varphi_{3},\sin\theta_{3}\sin\varphi_{3},\cos\theta_{3}\right),r_{011}=\left(\sin\theta_{4}\cos\varphi_{4},\sin\theta_{4}\sin\varphi_{4},\cos\theta_{4}\right), \end{array}$$

with angular parameters constrained by $\theta_{i}\in \left[0,\pi \right]$ and $\varphi_{i}\in \left[0,2\pi \right)$ for i=1,2,3,4. The parity-oblivious condition imposes the following nonlinear constraints:

(18) $$\left\{\begin{array}{cc} \; & \sin\theta_{1}\cos\varphi_{1}+\sin\theta_{4}\cos\varphi_{4}=\sin\theta_{2}\cos\varphi_{2}+\sin\theta_{3}\cos\varphi_{3},\\ \; & \sin\theta_{1}\sin\varphi_{1}+\sin\theta_{4}\sin\varphi_{4}=\sin\theta_{2}\sin\varphi_{2}+\sin\theta_{3}\sin\varphi_{3},\\ \; & \cos\theta_{1}+\cos\theta_{4}=\cos\theta_{2}+\cos\theta_{3}. \end{array}\right.$$

For the measurement operators, we parameterize the POVM measurements through their Bloch vectors

(19) $$m_{i}^{0}=\left(\sin\eta_{i}\cos\phi_{i},\sin\eta_{i}\sin\phi_{i},\cos\eta_{i}\right),\eta_{i}\in \left[0,\pi \right],\phi_{i}\in \left[0,2\pi \right),$$

where i=1,2,3. Under the conventional coordinate alignment, we let $\eta_{1}=0$, then $m_{1}^{0}=\left(1,0,0\right)$. This choice establishes the reference frame without loss of generality, simplifying the subsequent optimization problem. More precisely, the maximum expected success probability $E_{q}$ for the 3→1 PO-QRAC is formally characterized by the following constrained optimization problem:

(20) $$\begin{array}{c} \begin{array}{cc} \mathrm{Maximize}\text{:}\; & E_{q},\\ \mathrm{Subject}\;\mathrm{to}\text{:}\; & E_{q}=\frac{1}{2}+\frac{1}{24}(r_{000}\cdot m_{1}^{0}+r_{000}\cdot m_{2}^{0}+r_{000}\cdot m_{3}^{0}\\ \; & \;+r_{001}\cdot m_{1}^{0}+r_{001}\cdot m_{2}^{0}+r_{001}\cdot m_{3}^{1}\\ \; & \;+r_{010}\cdot m_{1}^{0}+r_{010}\cdot m_{2}^{1}+r_{010}\cdot m_{3}^{0}\\ \; & \;+r_{011}\cdot m_{1}^{0}+r_{011}\cdot m_{2}^{1}+r_{011}\cdot m_{3}^{1}),\\ \; & r_{000}+r_{011}=r_{001}+r_{010}. \end{array} \end{array}$$

Through numerical optimization using semidefinite programming, we determine that $E_{q}^{\max}$ = 0.7887. Under this configuration, where $m_{2}^{0}=\left(0,1,0\right)$, $m_{3}^{0}=\left(1,0,0\right)$, and

(21) $$r_{x}=\frac{\sqrt{3}}{3}\sum_{i=1,2,3}m_{i}^{{\left(-1\right)}^{x_{i}}},$$

the theoretical upper bound of $E_{q}$ is achieved. The three POVM measurements correspond to three mutually orthogonal pairs of antipodal Bloch vectors. This arrangement achieves the fundamental geometric limit for two-dimensional quantum systems. Specifically, in any qubit Hilbert space, the orthogonality-dimension complementarity principle dictates that no more than three mutually orthogonal vector pairs can coexist.

For n=4, the parity-oblivious constraint set $S_{4}$ comprises 11 elements, each corresponding to a distinct non-trivial parity function. Under the antipodal condition, the analysis reduces to considering only those parity constraints with elements of $S_{4}$ satisfying wt(S) is odd. We thus define a refined parity-oblivious constraint set $S_{4}^{\prime }$, specifically comprising 0111, 1011, 1101, and 1110. Then, the parity-oblivious constraint translates into solving the following system of equations:

(22) $$\begin{array}{c} r_{0000}+r_{0011}+r_{0101}+r_{0110}=r_{0111}+r_{0100}+r_{0010}+r_{0001}, \end{array}$$

(23) $$\begin{array}{c} r_{0000}+r_{0011}+r_{0100}+r_{0111}=r_{0110}+r_{0101}+r_{0010}+r_{0001}, \end{array}$$

(24) $$\begin{array}{c} r_{0000}+r_{0010}+r_{0101}+r_{0111}=r_{0110}+r_{0100}+r_{0011}+r_{0001}, \end{array}$$

(25) $$\begin{array}{c} r_{0000}+r_{0001}+r_{0110}+r_{0111}=r_{0101}+r_{0100}+r_{0011}+r_{0010}. \end{array}$$

**Figure 2:**
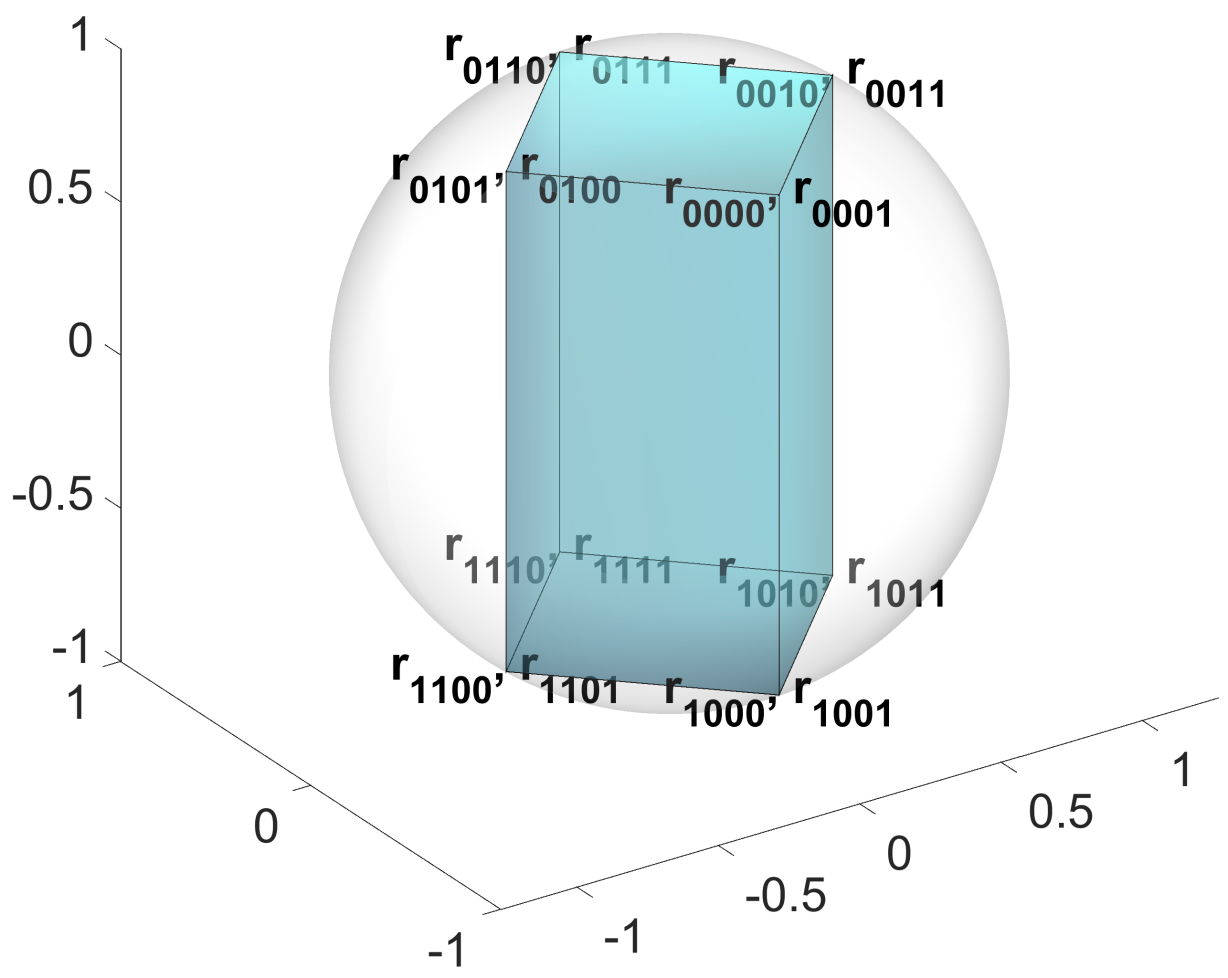
Geometric representation of encoded quantum states in the 4→1 PO-QRAC. The spatial distribution of Bloch vectors for the encoded states satisfies the symmetry constraints defined in Equations (26) and (27), forming the eight vertices of an equidiagonal parallelepiped on the Bloch sphere (rendered as a semitransparent gray sphere). This geometric configuration fundamentally differs from the tetrahedral arrangement characterizing the optimal 4→1 QRAC, highlighting the structural impact of parity-oblivious constraints on quantum state geometry.

Following algebraic simplification of the constraint equations (Equations (22)–(25)), we derive the reduced system:

(26) $$\begin{array}{cc} r_{0000}+r_{0111}=r_{0001}+r_{0110} & =r_{0010}+r_{0101}=r_{0011}+r_{0100}=k, \end{array}$$

(27) $$\begin{array}{cc} r_{0000}+r_{0011} & =r_{0001}+r_{0010}, \end{array}$$

where k denotes a fixed reference vector. Equations (26) and (27) reveal that the eight unit vectors geometrically constitute an equidiagonal parallelepiped, which is tangent to the unit Bloch sphere. $r_{0000}$, $r_{0010}$, $r_{0110}$, and $r_{0100}$ form a rectangle lying on a circle of the Bloch sphere, see Figure 2.

Under the above genetic conditions, we define the vector $r_{0000}$ as

(28) $$\begin{array}{cc} r_{0000} & =(\sin\theta_{0}\cos\varphi_{0},\sin\theta_{0}\sin\varphi_{0},\cos\theta_{0}), \end{array}$$

where $\theta_{0}\in \left[0,\pi \right]$ and $\varphi_{0}\in \left[0,2\pi \right)$. The other encoded quantum states are determined based on $r_{0000}$.

Given the fundamental geometric constraint that a two-dimensional Hilbert space permits at most three mutually orthogonal measurement bases, our protocol strategically duplicates one measurement basis to accommodate the fourth required setting in the 4→1 PO-QRAC optimization. Following the optimal QRAC configuration framework, we intentionally align the fourth measurement basis with one of the three existing orthogonal pairs. Without loss of generality, we implement the following Bloch vector assignments:

(29) $$\begin{array}{c} m_{1}^{0}=\left(0,0,1\right),m_{2}^{0}=\left(0,1,0\right), \end{array}$$

(30) $$\begin{array}{c} m_{3}^{0}=\left(1,0,0\right),m_{4}^{0}=\left(0,0,1\right). \end{array}$$

The expected success probability $E_{q}$ for the 4→1 PO-QRAC can be written as

(31) $$\begin{array}{c} E_{q}=\frac{1}{2}+\frac{1}{128}\sum_{x\in {\left\{0,1\right\}}^{4}}r_{x}\cdot \left(\sum_{i=1,2,3,4}m_{i}^{x_{i}}\right). \end{array}$$

**Figure 3:**
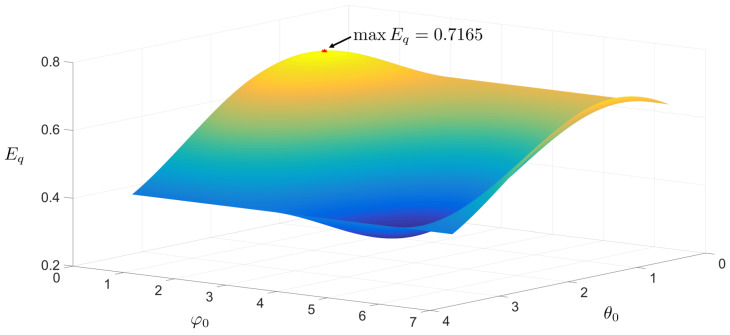
Relationship of the expected success probability $E_{q}$ on spherical coordinates $\theta_{0}$ and $\varphi_{0}$ for the 4→1 PO-QRAC. The maximum $E_{q}=0.7165$ (marked by the red datapoint) is achieved at $\theta_{0}=\frac{5\pi }{16}$ and $\varphi_{0}=\frac{\pi }{4}$.

By varying the spherical coordinate parameters $\theta_{0}$ and $\varphi_{0}$, we characterize the functional relationship between these angular variables and the expected success probability $E_{q}$ of the 4→1 PO-QRAC. Numerical optimization reveals a global maximum of $E_{q}=0.7165$ at $\theta_{0}=\frac{5\pi }{16}$ and $\varphi_{0}=\frac{\pi }{4}$, corresponding to a geometrically optimal Bloch vector configuration. The dependence of $E_{q}$ on $\theta_{0}$, and $\varphi_{0}$ is fully mapped in Figure 3.

The above result demonstrates an enhancement over the previously reported bound of $1/2+\sqrt{2}/8$ in [31], while rigorously satisfying the parity-obliviousness constraint. However, the result falls short of the theoretical upper bound $E_{q}=0.75$ for the 4→1 PO-QRAC reported in [32]. Achieving that bound in a qubit system would require constructing four mutually complementary measurements, yet any qubit admits at most three such measurements, corresponding to the three Pauli matrices. To reach $E_{q}=0.75$, one must upgrade the protocol to a four-dimensional Hilbert space, where the necessary observables can be built from the generalized Gell-Mann matrices. In that setting, the scheme becomes a 4→2 PO-QRAC and can attain the theoretical maximum.

## 5. Randomness Certification

Following the analytical determination of maximum success probabilities for n→1 PO-QRACs with n=2,3,4, we now investigate the certifiable randomness generated by quantum randomness expansion protocols based on this family of quantum access codes. The two conditions that must be met in order to generate fresh randomness are as follows:

1. Violation of a quantum witness, i.e., $E_{q}>E_{c}^{\max}$, where $E_{q}$ is the quantum expected success probability and $E_{c}^{\max}$ denotes the classical bound.
2. The min-entropy $H_{\infty }\left(B|X,Y\right)$ of the output bit must be strictly positive, i.e., $H_{\infty }\left(B|X,Y\right)>0$.

As demonstrated in our prior analyses, the maximum expected success probabilities of n→1 PO-QRACs for n=2,3,4 exceed their respective classical bounds, thereby satisfying the first criterion for randomness generation. We now characterize the parametric conditions under which the second critical requirement is fulfilled.

We now proceed to establish a lower bound on the min-entropy conditioned on the expected success probability $E_{q}$ of the n→1 PO-QRAC, which can be obtained by solving the following optimization problem:

(32) $$\begin{array}{c} \begin{array}{cc} \mathrm{Minimize}\text{:}\; & H_{\infty }\left(B|X,Y\right),\\ \mathrm{Subject}\;\mathrm{to}\text{:}\; & E_{q}=\frac{1}{n\cdot 2^{n}}\sum_{x\in {\left\{0,1\right\}}^{n}}\sum_{y=1}^{n}p\left(b=x_{y}|x,y\right),\\ \; & \forall S\in S_{n}:\;\sum_{x\in {\left\{0,1\right\}}^{n}}{\left(-1\right)}^{{⨁}_{i\in S}x_{i}}\rho_{x}=0. \end{array} \end{array}$$

Following Equation (2), the optimization problem can be reformulated as maximizing the conditional probability $p(b=x_{y}|x,y)$ for a given target expected success probability $E_{q}$. In the optimization problem, the set of achievable pairs $\left(E_{q},\max p\left(b=x_{y}|x,y\right)\right)$ forms a concave region. Consequently, the function *f* that returns the maximal value of $\max p(b=x_{y}|x,y)$ for a fixed $E_{q}$ coincides with the inverse mapping that returns the maximal $E_{q}$ for a fixed $\max p(b=x_{y}|x,y)$. We then derive $f^{-1}$ by solving the following optimization:

(33) $$\begin{array}{c} \begin{array}{cc} \mathrm{Maximize}\text{:}\; & E_{q}=\frac{1}{n\cdot 2^{n}}\sum_{x\in {\left\{0,1\right\}}^{n}}\sum_{y=1}^{n}p\left(b=x_{y}|x,y\right),\\ \mathrm{Subject}\;\mathrm{to}\text{:}\; & \max_{x,y}p\left(b=x_{y}|x,y\right)=p,\\ \; & \;\;\forall S\in S_{n}:\;\sum_{x\in {\left\{0,1\right\}}^{n}}{\left(-1\right)}^{{⨁}_{i\in S}x_{i}}\rho_{x}=0, \end{array} \end{array}$$

where *p* is the fixed maximum probability value.

For n=2, we define $m_{1}^{0}=\left(1,0,0\right)$ and $m_{2}^{0}=\left(\cos\alpha ,\sin\alpha ,0\right)$, 0⩽α⩽π. Without loss of generality, let p(b=1∣11,1)=p and set the corresponding Bloch vector $r_{11}=\left(-\cos\beta ,-\sin\beta ,0\right)$, where β=arccos(2p−1). To maximize the value of $E_{q}$, the optimal preparations for the remaining inputs are

(34) $$r_{01}=\frac{m_{1}^{0}+m_{2}^{1}}{\|m_{1}^{0}+m_{2}^{1}\|},r_{10}=\frac{m_{1}^{1}+m_{2}^{0}}{\|m_{1}^{1}+m_{2}^{0}\|}.$$

Finally, enforcing parity-obliviousness requires $r_{00}=-r_{11}$.

We derive the expected success probability $E_{q}$ as

(35) $$E_{q}=\frac{1}{2}+\frac{1}{8}\left(\sqrt{2-2\cos\alpha }+\left(2p-1\right)\left(1+\cos\alpha \right)+2\sqrt{p^{2}-p}\sin\alpha \right).$$

The inverse function $f^{-1}\left(p\right)$ is correspondingly given by

(36) $$f^{-1}\left(p\right)=\frac{1}{2}+\frac{1}{8}\left(\sqrt{2-2\cos\alpha_{p}}+\left(2p-1\right)\left(1+\cos\alpha_{p}\right)+2\sqrt{p-p^{2}}\sin\alpha_{p}\right),$$

where $\alpha_{p}$ denotes the critical point that extremizes $f^{-1}$ with respect to α and $0⩽\alpha_{p}⩽\pi$.

**Figure 4:**
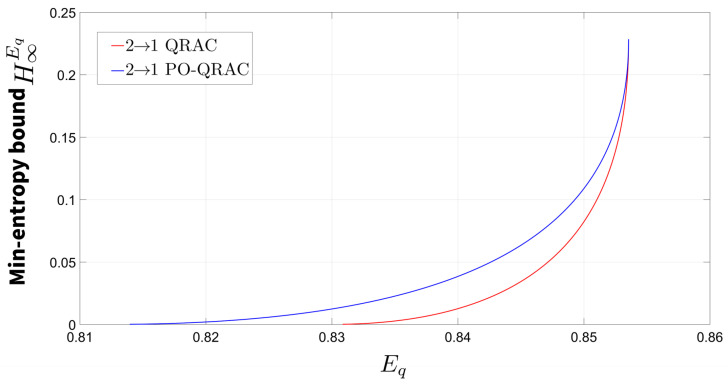
The relationship between the min-entropy bound $H_{\infty }^{E_{q}}$ and the expected success probability $E_{q}$ for the 2→1 PO-QRAC. The red curve represents the randomness expansion protocol based on 2→1 QRAC, while the blue curve corresponds to the enhanced protocol utilizing 2→1 PO-QRAC. Comparative analysis demonstrates that the latter achieves a broader certifiable randomness range. Notably, when the min-entropy assumes positive values, the $E_{q}$ consistently surpasses its classical counterpart $E_{c}=3/4$.

The relationship between the min-entropy bound $H_{\infty }^{E_{q}}$ and the expected success probability $E_{q}$ for the 2→1 PO-QRAC is plotted in Figure 4. The result shows that the integration of parity-oblivious constraints into the randomness expansion protocol enables the generation of certifiable randomness at an enhanced rate.

For the case of n=3, to ensure $m_{1}^{0}m_{2}^{0}=m_{1}^{0}m_{3}^{0}=m_{2}^{0}m_{3}^{0}$, the measurements are defined as

(37) $$\begin{array}{cc} m_{1}^{0} & =(1,0,0), \end{array}$$

(38) $$\begin{array}{cc} m_{2}^{0} & =(\cos2\alpha ,\sin2\alpha ,0), \end{array}$$

(39) $$\begin{array}{cc} m_{3}^{0} & =(\cos\alpha \sin\beta ,\sin\alpha \sin\beta ,\cos\beta ), \end{array}$$

where 0⩽α⩽π/2, −π/2⩽β⩽π/2. Assuming that p(b=1∣111,3)=p, the states can be defined as

(40) $$\begin{array}{cc} \; & r_{111}=\left(-\cos\alpha \sin\left(\beta +\gamma \right),-\sin\alpha \sin\left(\beta +\gamma \right),-\cos\left(\beta +\gamma \right)\right), \end{array}$$

(41) $$\begin{array}{cc} \; & r_{000}=-r_{111}, \end{array}$$

(42) $$\begin{array}{cc} \; & r_{x\neq 000,111}=\frac{m_{1}^{x_{1}}+m_{2}^{x_{2}}+m_{3}^{x_{3}}}{\|m_{1}^{x_{1}}+m_{2}^{x_{2}}+m_{3}^{x_{3}}\|}, \end{array}$$

where $x\in {\left\{0,1\right\}}^{3}$. We can derive the inverse function $f^{-1}\left(p\right)$ as

(43) $$\begin{array}{c} \begin{array}{cc} f^{-1}\left(p\right)= & \frac{1}{2}+\frac{3}{8}\sqrt{3-2\cos2\alpha_{p}}\\ \; & +\frac{1}{24}[\left(2p-1\right)\left(1+2\cos^{2}\alpha_{p}-2\right)+2\sqrt{\left(p-p^{2}\right)\left(5-4\cos^{2}\alpha_{p}-\frac{1}{\cos^{2}\alpha_{p}}\right)}], \end{array} \end{array}$$

where $\alpha_{p}$ denotes the extreme point of $f^{-1}$ with respect to α and $0⩽\alpha_{p}⩽\pi$. The optimization process must strictly maintain the balance equation $r_{000}+r_{011}=r_{010}+r_{001}$.

**Figure 5:**
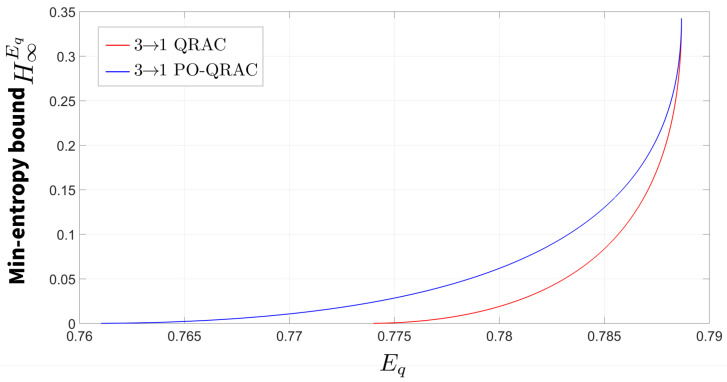
The relationship between the min-entropy bound $H_{\infty }^{E_{q}}$ and the expected success probability $E_{q}$ for the 3→1 PO-QRAC. The red curve represents the randomness expansion protocol based on 3→1 QRAC, while the blue curve corresponds to the enhanced protocol utilizing 3→1 PO-QRAC. Comparative analysis demonstrates that the latter achieves a broader certifiable randomness range. Notably, when the min-entropy assumes positive values, the $E_{q}$ consistently surpasses its classical counterpart $E_{c}=2/3$.

The relationship between the min-entropy bound $H_{\infty }^{E_{q}}$ and the expected success probability $E_{q}$ for the 3→1 PO-QRAC is plotted in Figure 5. Our analysis reveals that the quantum randomness expansion protocol based on the 3→1 PO-QRAC achieves a higher certified randomness generation rate compared to its 2→1 PO-QRAC counterpart.

The proposed 3→1 PO-QRAC-based randomness expansion protocol achieves a higher certified min-entropy compared to conventional QRAC implementations at identical quantum witness values $E_{q}$. Additionally, the results presented in Section 4 demonstrate that the 3→1 PO-QRAC achieves the maximum quantum success probability among all investigated configurations. This optimal performance simultaneously maximizes the certifiable min-entropy. Crucially, SDI randomness expansion protocols utilizing 3→1 PO-QRAC outperform both 2→1 and 4→1 variants in entropy generation efficiency, establishing it as the optimal protocol configuration for quantum randomness expansion under parity-oblivious constraints.

## 6. Conclusions

We have investigated randomness-expansion protocols based on the n→1 PO-QRACs. After introducing the protocol model, we derived tight upper bounds on the maximum success probability in both the classical and quantum settings. Our analysis shows that for n=2,3,4, the PO-QRAC always outperforms its classical counterpart, making it a viable primitive for SDI randomness expansion. Remarkably, in the case of n=4, the quantum bound under the parity-oblivious constraint even exceeds the limit reported in [31]. However, for n>3, no two-dimensional PO-QRAC can attain the theoretical maximum of 0.75 derived by [32], since that bound demands the existence of *n* mutually unbiased bases in a qubit system, which is impossible. Finally, we established an analytic relation between the quantum witness $E_{q}$ and the certifiable randomness (min-entropy) in these protocols. Numerically, we find that for n=2,3, PO-QRAC-based expansion certifies strictly more randomness than standard QRAC-based schemes at the same $E_{q}$ value, demonstrating the advantage of enforcing parity obliviousness. The SDI randomness expansion protocol constructed using the 3→1 PO-QRAC represents the optimal implementation framework for quantum randomness expansion under parity-oblivious constraints.

## Data Availability

Data are contained within the article.
